# Clarifying Mendelian vs non-Mendelian inheritance

**DOI:** 10.1093/genetics/iyae078

**Published:** 2024-05-28

**Authors:** Susan Strome, Needhi Bhalla, Rohinton Kamakaka, Upasna Sharma, William Sullivan

**Affiliations:** Department of Molecular, Cell, and Developmental Biology, University of California Santa Cruz, Santa Cruz, CA 95064, USA; Department of Molecular, Cell, and Developmental Biology, University of California Santa Cruz, Santa Cruz, CA 95064, USA; Department of Molecular, Cell, and Developmental Biology, University of California Santa Cruz, Santa Cruz, CA 95064, USA; Department of Molecular, Cell, and Developmental Biology, University of California Santa Cruz, Santa Cruz, CA 95064, USA; Department of Molecular, Cell, and Developmental Biology, University of California Santa Cruz, Santa Cruz, CA 95064, USA

**Keywords:** genetics, Mendel's laws, Mendelian inheritance, genotypic and phenotypic ratios

## Abstract

Gregor Mendel developed the principles of segregation and independent assortment in the mid-1800s based on his detailed analysis of several traits in pea plants. Those principles, now called Mendel's laws, in fact, explain the behavior of genes and alleles during meiosis and are now understood to underlie “Mendelian inheritance” of a wide range of traits and diseases across organisms. When asked to give examples of inheritance that do NOT follow Mendel's laws, in other words, examples of non-Mendelian inheritance, students sometimes list incomplete dominance, codominance, multiple alleles, sex-linked traits, and multigene traits and cite as their sources the Khan Academy, Wikipedia, and other online sites. Against this background, the goals of this Perspective are to (1) explain to students, healthcare workers, and other stakeholders why the examples above, in fact, display Mendelian inheritance, as they obey Mendel's laws of segregation and independent assortment, even though they do not produce classic Mendelian phenotypic ratios and (2) urge individuals with an intimate knowledge of genetic principles to monitor the accuracy of learning resources and work with us and those resources to correct information that is misleading.

## The motivation for this Perspective

We were motivated to develop this Perspective by an anecdotal report that students were confused about the basis for categorizing inheritance as Mendelian vs non-Mendelian. That report prompted us to survey students in undergraduate classes at several institutions on the categorization of various genetic phenomena (including complete dominance, incomplete dominance, codominance, multiple alleles, sex-linked traits, multigene traits, mitochondrial genes, and epigenetics) as displaying Mendelian or non-Mendelian inheritance. Students were also asked to cite their source(s) of information. An overwhelming majority (92%) of the 461 students surveyed correctly classified completely dominant and recessive traits as following the rules of Mendelian inheritance. Disturbingly, a much smaller percentage of students across the 3 institutions (25–52% depending on the genetic example) correctly classified incomplete dominance, codominance, multiple alleles, sex-linked traits, and multigene traits as Mendelian, and some students who misclassified those as non-Mendelian cited the Khan Academy, Wikipedia, and other online sites as their sources of information. Indeed, both the Khan Academy and Wikipedia list incomplete dominance, codominance, multiple alleles, and multigene traits as non-Mendelian. Why this confusion? As developed below, we propose that the confusion stems from whether inheritance is categorized based on genotypic ratios or phenotypic ratios. We argue that inheritance should be classified as Mendelian or non-Mendelian based on genotypic, not phenotypic, ratios, since phenotypic ratios reflect the relationship between alleles rather than the mode of inheritance.

## Introduction: what we learned from Mendel's work

Mendel developed the principles of segregation, independent assortment, and dominance based on his studies of 7 traits in peas, including flower color, pea color, and pea shape (Mendel 1865-1866; Bateson 1909; Sturtevant 1965). The pea traits that Mendel focused on were determined by single genes, each of which had 2 alternativ alleles, 1 fully dominant and 1 fully recessive. We now know that most traits in organisms are determined by multiple genes, that individual genes often have multiple alleles, and that some alleles display variations in dominance, including incomplete dominance and codominance. The involvement of multiple genes, the possibility of multiple alleles, and unusual dominance relationships between different alleles do not influence how the 2 parental alleles of each gene are sorted into haploid gametes that fuse to form diploid offspring. Thus, the resulting offspring traits, whether in peas or other organisms, result from parental alleles that follow Mendel's laws of segregation and independent assortment. Because of this, the inheritance patterns of such traits are often discussed in Genetics textbooks as variations or extensions of Mendelian inheritance (e.g. Russell 2010; Snustad and Simmons 2016; Pierce 2020; Brooker 2021; Goldberg *et al.* 2021). Because Mendel's law of dominance does not impact how alleles are inherited but instead how they contribute to phenotypes in offspring, this Perspective focuses on Mendel's laws of segregation and independent assortment to classify inheritance as Mendelian or non-Mendelian. For a recent and helpful discussion of dominance and recessiveness, see Zschocke *et al.* (2023).

**Fig. 1.**
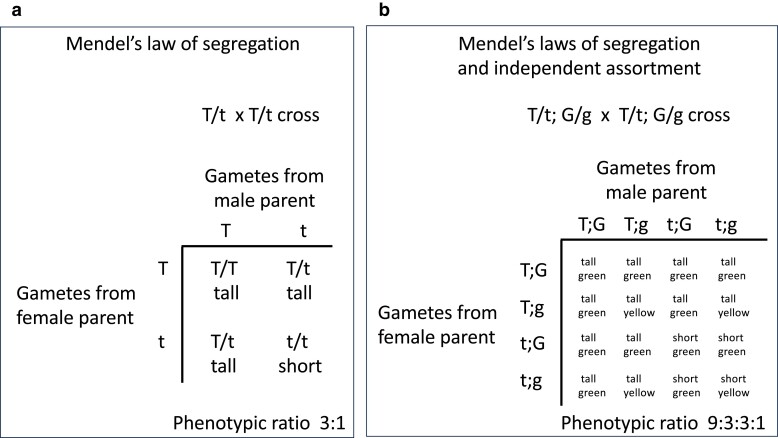
Punnett squares illustrating Mendel's laws of segregation and independent assortment. These Punnett squares show the genotypes of gametes produced by each heterozygous parent during meiosis (on the sides of the squares) and the genotypes and phenotypes of the offspring that result from the various unions of gametes (in the center of the squares). a, b) According to Mendel's law of segregation, the 2 alleles of a gene are segregated to different gametes during meiosis and then united at random, 1 from each parent, at fertilization. b) According to Mendel's law of independent assortment, when 2 genes are on different chromosomes (i.e. unlinked), they segregate independently. Only offspring phenotypes are shown. In these examples, T is the dominant allele that causes pea plants to be tall and G is the dominant allele that causes pea pods to be green.

**Fig. 2.**
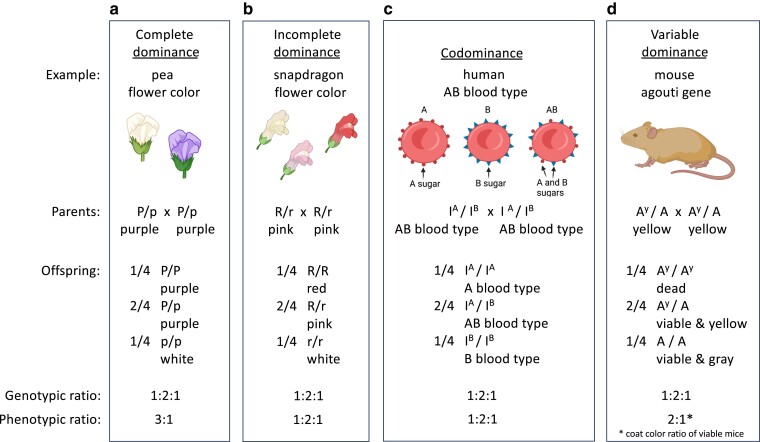
Examples of complete dominance, incomplete dominance, codominance, and variable dominance. From crosses of heterozygous parents, all display a Mendelian genotypic ratio of 1:2:1 among offspring and show Mendelian inheritance. a) Complete dominance results in a 3:1 phenotypic ratio, as illustrated in Fig. 1a. b) Incomplete dominance, c) codominance, and d) variable dominance cause altered phenotypic ratios as a result of the action of the gene products in offspring.

The molecular basis of segregation and independent assortment of alleles lies in the behavior of chromosomes during meiosis. Mendel did not know about chromosomes and meiosis, but the traits he studied and the principles he formulated beautifully and presciently predicted chromosome behavior in meiosis. In diploid organisms, like peas, the 2 versions of each chromosome, 1 from each parent, segregate into the haploid gametes during meiosis. The union of 2 gametes at fertilization restores diploidy, with offspring inheriting 1 version of each chromosome from each parent. The pea traits or “phenotypes” that Mendel studied are dictated by genes that reside on chromosomes. The alleles of those genes segregate from each other into gametes during meiosis in the parents and are united at random in the offspring (Fig. 1a). This forms the basis for Mendel's law of segregation. When different genes are on different chromosomes, as most of the pea genes studied by Mendel are, they behave independently of one another (Fig. 1b). This forms the basis for Mendel's law of independent assortment. The power of our now-deep understanding of meiosis is the ability to predict offspring genotypes and phenotypes and their ratios that parents will produce. Indeed, in Mendel's experiments, he observed that crossing true-breeding plants with purple flowers (P/P) and true-breeding plants with white flowers (p/p) generated all plants with purple flowers (P/p); crossing those P/p plants with one another generated 1/4 P/P purple-flower plants, 2/4 P/p purple-flower plants, and 1/4 p/p white-flower plants (Fig. 2a).

Mendel's published studies (Mendel 1865-1866) focused on 2 versions of each of the pea plant traits he studied. For example, for flower color, the dominant P allele led to purple flowers, and the recessive p allele led to white flowers. We now understand the molecular biology for some of those traits and the basis for some of them, such as flower color, to display variations beyond those studied by Mendel (Ellis *et al.* 2011). Notably, Mendel's focus on single-gene, 2-allele traits derived from clear dominant and recessive relationships enabled him to develop the laws of segregation and independent assortment. It is a tribute to Mendel that he accurately deduced the transmission of genes and alleles from parents to offspring based on analyzing the *phenotypes* of parents and offspring. That has led to some confusion about whether genotypic ratios or phenotypic ratios should dictate whether inheritance is described as Mendelian or non-Mendelian. In this Perspective, we discuss the reasoning behind our opinion that Mendelian inheritance should always be distinguished based on genotypic ratios that derive from segregation and independent assortment during meiosis.

In addition to Mendel's laws of segregation and independent assortment, Mendel's work led to the recognition of 2 additional features of inheritance: that alleles are passed unaltered through multiple generations (even through generations in which a recessive allele does not display its phenotype due to the presence of a dominant allele), and that the phenotypes of individuals are dictated by their genotypes. We discuss how some examples of non-Mendelian inheritance violate these inheritance features.

## Extensions of Mendel's laws and variations in phenotypic ratios

**Fig. 3.**
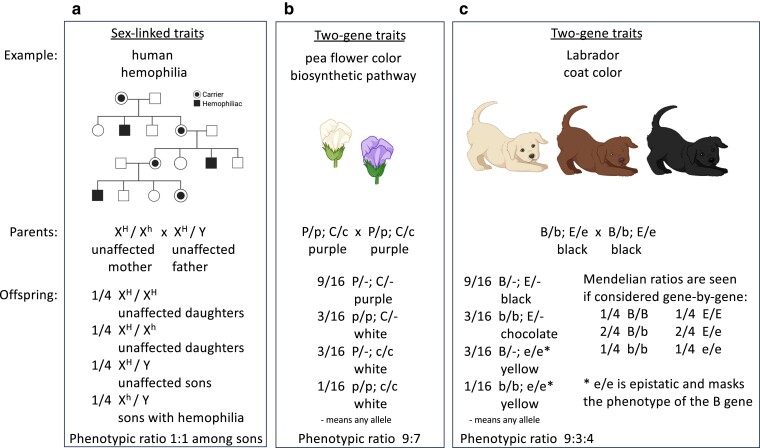
Examples of sex-linked traits and 2-gene traits. Both display Mendelian genotypic ratios and show Mendelian inheritance patterns. a) The sample pedigree shows the inheritance of hemophilia in humans (filled symbols). Because the mutant hemophilia allele h is recessive and resides on the X chromosome, the disease phenotype shows a sex bias. From the parents shown, sons (with only 1 X chromosome) have a 50:50 chance of being affected, while daughters (with 2 X chromosomes) will not be affected. b) Purple flower color in peas requires the synthesis of anthocyanin. In this example, the unlinked P gene and C gene function at different steps in the anthocyanin biosynthetic pathway. From crosses of heterozygous parents, the absence of either gene product (in p/p or c/c plants) results in white flowers in offspring and the unusual phenotypic ratio of 9 purple-flower : 7 white-flower plants. c) Labrador dog coat color is controlled by 2 unlinked genes: the E gene for the production of black pigment and the B gene for dense deposition of black pigment in dog hairs. From crosses of heterozygous parents, each gene displays a Mendelian genotypic ratio of 1:2:1 among offspring. However, the offspring display an unusual phenotypic ratio of 9 black : 3 chocolate : 4 yellow dogs as a result of the action of the E and B gene products and epistasis; among offspring, e/e is epistatic and masks the phenotype of the B gene.

In Mendel's studies of pea traits, from crosses between heterozygous parents (e.g. P/p crossed with P/p), the offspring showed a 1:2:1 genotypic ratio (1 P/P : 2 P/p : 1 p/p) and a 3:1 phenotypic ratio (3 purple:1 white; Fig. 2a). The relationships between alleles that underlie variations in dominance, the existence of more than 2 alleles of a gene, sex-linked traits, and multigene traits result in altered phenotypic ratios (as shown in Figs. 2 and 3); nevertheless, the 2 alleles of each gene in an individual still follow the law of segregation during meiosis and still produce Mendelian genotypic ratios in offspring, thus displaying Mendelian inheritance. This can be seen in Punnett squares (Fig. 1), branch diagrams, and pedigrees (Fig. 3a) showing the passage of alleles from parents to offspring. Below are examples of each, taken from and developed more fully in the textbook “Genetics: From Genes to Genomes” 7th edition by Goldberg *et al.* (2021).

### Incomplete dominance

In cases of incomplete dominance, the phenotype of a heterozygote with 2 different alleles of a gene is intermediate between the phenotypes of the homozygotes. An example is flower color in snap dragons (Fig. 2b). Homozygotes for an allele that causes the production of red pigment have red flowers. Homozygotes for an allele that does not produce pigment have white flowers. Heterozygotes with a red-producing allele and a white allele are pink, which is intermediate in color between red and white. This is because in heterozygotes, only 1 allele produces pigment, which is enough to make flowers pink but not enough to make them red. (In the case of pea flower color where the P allele shows complete dominance, in heterozygous P/p plants, 1 P allele is sufficient to make the flowers purple.) When plants with pink flowers are crossed together, the next generation of plants display all 3 flower colors in the ratio of 1 red : 2 pink : 1 white. Thus, from crossing heterozygous snapdragons, both the genotypic and the phenotypic ratios are 1:2:1. The alleles that control snapdragon flower color segregate properly to gametes during meiosis and thus follow Mendel's law of segregation. The phenotypic ratio is different from what Mendel saw for pea flower color, because the downstream relationship between the 2 alleles of the flower-color gene in heterozygotes and the pigment produced differ between peas and snap dragons.

### Codominance

In cases of codominance, the phenotypes caused by 2 different alleles of the same gene are *both* expressed. An example is the human AB blood type (Fig. 2c). The human blood type is determined by the I gene. The A allele of the gene (called I^A^) causes red blood cells to have the A sugar on their cell surface. The B allele (I^B^) causes red blood cells to have the B sugar on their surface. The I^A^ and I^B^ alleles are codominant: I^A^/I^B^ individuals have *both* the A and the B sugars on their red blood cells and therefore have an AB blood type. Thus, from a cross of I^A^/I^B^ heterozygotes, both the genotypic ratio and the phenotypic ratio observed in offspring are 1:2:1. Once again, although the phenotypic ratio is not the 3:1 of Mendel's pea flowers, the 2 alleles of the I gene in an individual segregate according to Mendel's law. It is the relationship between the gene products from the alleles that produces variations in phenotypes, particularly in heterozygotes.

### Multiple alleles

Many genes have more than 2 alleles in a population of individuals. An example is the agouti gene in mice, which has at least 19 alleles (Bultman *et al.* 1992). Importantly, individual mice have only 2 alleles, and those 2 alleles (whatever those 2 alleles may be) are transmitted in a Mendelian fashion. The alleles display a dominance series; for example, the A allele is dominant to the a^t^ allele and the a allele, and the a^t^ allele is dominant to the a allele. The agouti locus regulates the distribution of yellow and black pigment in mouse fur on the back and belly. The various combinations of alleles cause mice to have coat colors that range from black to gray to yellow, sometimes with different coloration on the back and belly. The 2 alleles from each parent are transmitted to offspring exactly as Mendel's law predicts. The difference in coat color among offspring is simply the consequence of the particular dominance relationship between the molecular products of the 2 alleles present in an individual mouse.

The A^Y^ allele of the mouse agouti gene illustrates an additional lesson about genes and alleles. The A^Y^ allele in heterozygotes, in combination with any of the other agouti alleles (e.g. A^Y^/A), causes mice to have yellow fur, while the A^Y^ allele in homozygotes (A^Y^/A^Y^) causes mice to die during gestation (Fig. 2d). Thus, the A^Y^ allele has a dominant effect on coat color and a recessive effect on viability. This underscores that the dominant–recessive relationship of 2 alleles of a gene depends on the phenotype being analyzed. Importantly, from a cross of A^Y^/A parents, the genotypic ratio in embryos is 1:2:1 due to Mendelian segregation, but the lethality of A^Y^/A^Y^ embryos during in utero development skews the coat-color phenotypic ratio observed in viable offspring to 2:1.

An appreciation of the diversity of allele types and interactions is emerging from the isolation of many alleles of heavily studied genes (e.g. the mouse agouti gene; Bultman *et al.* 1992) and the now-common use of high-throughput genome sequencing in developmental and medical genetics (Zschocke *et al.* 2023). These and a number of earlier studies make clear that allele assignments may be more complicated than simply dominant or recessive, and that phenotypes are dictated by often-complex contributions of multiple allele pairs. These unusual relationships highlight the need for an unambiguous and clear understanding of the underlying Mendelian inheritance principles by scientists and especially health professionals.

### Sex-linked traits

Sex-linked traits in organisms commonly discussed in genetics courses, namely humans and fruit flies, deal with genes on the X chromosome and, less frequently, genes on the Y chromosome. Two examples are red-green color blindness and some forms of hemophilia in humans. The genes for these 2 traits reside on the X chromosome. The phenotypes caused by mutant alleles of the genes are more commonly observed in males with a single X chromosome than in females with 2 X chromosomes. Considering hemophilia (Fig. 3a), if H is the dominant normal allele and h is a recessive mutant allele that causes hemophilia, females that are X^H^/X^H^ or X^H^/X^h^ will not have hemophilia, while females that are X^h^/X^h^ and males that are X^h^/Y will have hemophilia. This results in interesting phenotypic ratios of affected offspring in pedigrees. For example, an unaffected father (X^H^/Y) and an unaffected heterozygous mother (X^H^/X^h^) can have some affected sons (X^h^/Y) and some unaffected sons (X^H^/Y), but none of their daughters (X^H^/X^h^ and X^H^/X^H^) will be affected. Since the X and Y chromosomes and their resident genes segregate from each other during meiosis, X- and Y-linked traits display Mendelian inheritance.

### Two-gene traits

When 2 unlinked genes regulate *different* traits, from doubly heterozygous parents, offspring display a 9:3:3:1 phenotypic ratio, as shown by Mendel in his dihybrid crosses (Fig. 1b). Deviations from this 9:3:3:1 ratio are observed when 2 unlinked genes regulate the *same* trait. Examples are genes that function in a pathway, such as a biosynthetic pathway, to produce a compound. We now understand that purple pea flowers require synthesis of the pigment anthocyanin via a biosynthetic pathway. Mutations that impair any step of that pathway, when homozygous, cause pea plants to have white flowers. Let us consider 2 steps in the pathway controlled by genes on different chromosomes (i.e. unlinked genes); 1 step is catalyzed by products of the P gene and the other by products of the C gene (see Miko 2008). In plants homozygous for a mutant allele of either gene (e.g. p/p or c/c), the resulting flowers are white. From a cross between pea plants heterozygous for both genes (i.e. P/p; C/c), the offspring plants would display a ratio of 9 purple-flower : 7 white-flower plants. The alleles for each gene segregate according to Mendel's law, giving a 1:2:1 genotypic ratio and a 3:1 purple:white phenotypic ratio, as observed by Mendel for the P gene alone. The 9:7 phenotypic ratio from crossing P/p; C/c plants arises because offspring homozygous for a mutant allele of either gene (e.g. p/p or c/c) produce white flowers (Fig. 3b). This example demonstrates that when 2 unlinked genes influence a single trait, Mendel's laws of segregation and independent assortment commonly result in phenotypic ratios that diverge from the classic 9:3:3:1.

Another example of genes acting in a pathway is coat color in Labrador retrievers, which relies on the E gene for the production of the black pigment eumelanin and the B gene for dense deposition of eumelanin in dog hairs (Fig. 3c). Labradors that produce eumelanin and deposit it densely are black. Labradors that produce eumelanin but cannot deposit it densely are brown or chocolate in color. Labradors that cannot produce eumelanin are yellow. The E and B genes are unlinked, and the alleles of both genes are transmitted in Mendelian fashion; when considered as single genes, offspring from heterozygous parents display a 1:2:1 genotypic ratio. It is the different combinations of alleles of the 2 genes that determine the 3 different coat-color phenotypes. This example illustrates a phenomenon termed “epistasis,” in which mutations in 1 gene mask the effects of a second gene (Miko 2008). In Labradors, the inability to produce eumelanin masks our seeing whether eumelanin can be deposited; this shifts the phenotypic ratio from 9:3:3:1 to 9:3:4 (Fig. 3c). Epistasis is sometimes mistakenly considered to be an example of non-Mendelian inheritance, but the individual alleles involved follow the laws of segregation and independent assortment perfectly and thus display Mendelian inheritance.

Many human traits such as height and skin color and conditions such as schizophrenia and autism are determined by numerous genes and are often classified as quantitative or continuous traits. Particularly when the number and identity of the genes involved are not known, inheritance patterns of these polygenic traits and conditions are difficult to predict. Furthermore, the phenotypes associated with many of these traits and conditions may be influenced by external factors including the environment. Nevertheless, the inheritance of alleles of these genes follow Mendel's laws of segregation and independent assortment.

### Linkage

The above examples illustrate the behavior of alleles of genes that reside on different chromosomes. Genes that are on the same chromosome (i.e. linked) violate Mendel's law of independent assortment due to physical linkage. Nevertheless, they display patterns of inheritance that can be predicted once the genes have been genetically mapped with relation to one another and the frequency of recombination between them has been quantified. Thus, even linked genes display Mendelian inheritance.

## Some examples of non-Mendelian inheritance

Genes that reside in the genomes of mitochondria and chloroplasts display non-Mendelian inheritance. The segregation of such non-nuclear organelles and the alleles in their genomes is thought to be largely random during the meiotic divisions. In many organisms and in most animals, these organelles and their resident genes are inherited by offspring mainly from the female parent (e.g. Giles *et al.* 1980; Sato and Sato 2013). Because organelle transmission does not follow Mendel's laws, the genes in organelles do not display Mendelian inheritance.

Epigenetic inheritance provides another example of non-Mendelian inheritance. Epigenetics refers to the regulation of gene expression and development by factors beyond the DNA sequence. The most heavily studied epigenetic regulators are DNA methylation, histone modifications, and small RNAs (reviewed in Allis *et al.* 2015). These regulators participate in such phenomena as genomic imprinting, paramutation, creation of epialleles, and RNA interference (Heard and Martienssen 2014; Fitz-James and Cavalli 2022; Yoosefzadeh Najafabadi *et al.* 2023 ). Epigenetic marking of genes by DNA methylation and histone modifications is subject to removal and addition in each generation in response to developmental cues and environmental influences, unlike the underlying DNA sequence. Because epigenetic marking is not passed unaltered through multiple generations, inheritance of the marks is considered non-Mendelian.

Maternal-effect genetics describes situations in which the phenotypes of individuals are dictated not by their genotypes but by the genotype of their mother. This is exemplified by the inheritance of snail coiling patterns (Sturtevant 1923) and by maternal control of early embryonic development in fruit flies (e.g. Nüsslein-Volhard *et al.* 1987). The logic behind this is that the maternal genotype dictates which gene products are packaged into oocytes, and those gene products dictate events in early embryos until embryos start generating gene products from their own genome (when zygotic transcription turns on). Because offspring development depends on the maternal genotype instead of the offspring's genotype, maternal-effect inheritance is considered non-Mendelian.

Interestingly, there are biological phenomena in which Mendelian inheritance of nuclear genes is distorted such that some alleles are preferentially transmitted to offspring. These phenomena, called “meiotic drive,” can act at different stages of gamete production and function to deliberately skew allele inheritance (Srinivasa and Zanders 2020). For example, in some maize plants, there are loci of repetitive DNA that cause heterochromatic “knobs,” so named because of their cytological appearance (McClintock 1930). Chromosomes with knobs can be homozygous, heterozygous, or absent in plants. When knob-bearing chromosomes are heterozygous in plants that also have a chromosome called “abnormal chromosome 10” (Ab10), knob chromosomes are preferentially segregated to the ovule, the sole gamete produced by female meiosis, with the other products of meiosis being discarded as polar bodies (Dawe 2022). Thus, more than 70% rather than the expected 50% of ovules carry knob chromosomes and their linked alleles, a clear distortion of Mendel's law of segregation (Rhoades 1942).

Some other versions of meiotic drive act after meiotic chromosome segregation; these include the Segregation Distorter locus in fruit flies (Larracuente and Presgraves 2012) and the t-haplotype in mice (Herrmann *et al.* 1999). In both of these examples, sperm that do not carry the locus associated with drive fail to develop properly, preventing their ability to participate in fertilization. Thus, sperm with the drive locus fertilize more eggs and produce more offspring than sperm without the drive locus. Meiotic drive phenomena provide fascinating molecular and evolutionary insights into how specific loci use “selfish” mechanisms to produce biased allele inheritance, in clear violation of Mendel's laws.

## The importance of understanding Mendelian inheritance

The main message of this Perspective is that categorizing patterns of inheritance as Mendelian or non-Mendelian should be based on genotypic ratios, not phenotypic ratios, in offspring. Genotypic ratios reflect the segregation and independent assortment of alleles during meiosis in parents, which are the underpinnings of Mendel's laws, while phenotypic ratios reflect the modes of action of the gene products and the relationships between alleles in offspring. Categorizing patterns of inheritance as Mendelian or non-Mendelian is not strictly an issue of semantics, but instead is based on an understanding of the various genetic situations that follow Mendel's laws but may produce unusual phenotypes in offspring. Textbooks describe these situations as extensions or variations of Mendelian inheritance (e.g. Russell 2010; Snustad and Simmons 2016; Pierce 2020; Brooker 2021; Goldberg *et al.* 2021). This Perspective makes clear why they are *not* violations of Mendelian inheritance.

Social media and online sites such as the Khan Academy and Wikipedia are valued and appreciated for providing accessible information quickly and efficiently and being receptive to input from the community. However, given their reliance on volunteers and crowdsourcing, they may provide incorrect or biased information. Therefore, community input should include experts monitoring the accuracy of the information and clarifying information that is misleading. Health care is 1 arena in which having access to accurate and easy-to-understand information is crucial. Primary-care providers must understand and be able to explain to patients and their families basic genetic concepts that underlie conditions and diseases, to help them make informed decisions (Houwink *et al.* 2011). Other stakeholders who need accurate online genetic information include anthropologists, ecologists, forensic pathologists, conservation biologists, commercial plant and animal breeders, and individuals involved in captive breeding programs for endangered species. Furthermore, to avoid confusion, students need accurate online information on basic Mendelian concepts that reinforces textbook and classroom information. Understanding the fundamental difference between genotypic and phenotypic ratios is essential to fully appreciate the extent to which specific traits are genetically determined and for informed discussions of more complex, sociopolitically constructed phenomena, such as race and gender, that move away from genetic determinism (Donovan, Weindling, *et al.* 2024; Donovan, Syed, *et al.* 2024; Duncan *et al.* 2024).

The authors of this Perspective plan to work with the Khan Academy, Wikipedia, and other online sites to clarify Mendelian vs non-Mendelian inheritance. We urge informed community members to join this effort and invite them to contact us with strategy and coordination ideas.
